# Vulnerability of Syrian refugees in Lebanon to COVID-19: quantitative insights

**DOI:** 10.1186/s13031-021-00349-6

**Published:** 2021-03-05

**Authors:** Fouad M. Fouad, Stephen J. McCall, Houssein Ayoub, Laith J. Abu-Raddad, Ghina R. Mumtaz

**Affiliations:** 1 Department of Epidemiology and Population Health, Faculty of Health Sciences, American University of Beirut, P.O.Box 11-0236, Riad El Solh, Beirut, 1107 2020 Lebanon; 2 Center for Research on Population and Health, Faculty of Health Sciences, American University of Beirut, Beirut, Lebanon; 3 Department of Mathematics, Statistics, and Physics, Qatar University, Doha, Qatar; 4 Infectious Disease Epidemiology Group, Weill Cornell Medical College – Qatar, Cornell University, Qatar Foundation - Education City, Doha, Qatar; 5 Department of Healthcare Policy and Research, Weill Cornell Medical College, Cornell University, New York, NY USA

**Keywords:** Refugee, Syria, COVID-19, Epidemic, Vulnerability

## Abstract

Lebanon, a middle-income country with ongoing political turmoil, unstable economic situation, and a fragmented and under-resourced health system, hosts about one million Syrian refugees since 2011. While the country is currently experiencing substantial COVID-19 epidemic spread, no outbreaks have been reported yet among Syrian refugees. However, testing of this population remains limited and exposure levels are high given dire living conditions and close interaction with the host community. Here, we use quantitative insights of transmission dynamics to outline risk and contextual factors that may modulate vulnerability of Syrian refugees in Lebanon to potentially large COVID-19 epidemics.

Syrian refugees live in close contact with the host community, and their living conditions are favorable for epidemic spread. We found that the high levels of crowding within Syrian refugee households and among those in informal tented settlements, the inadequate water supply and sanitation, limited use of masks, inadequate access to health care, and inadequate community awareness levels are vulnerability factors that directly impact important parameters of transmission dynamics, leading to larger epidemic scale. Poverty, stigma, and fear of legal consequences are contextual factors that further exacerbate this vulnerability. The relatively high prevalence of non-communicable diseases in this population could also affect the severity of the disease among those infected. Mathematical modeling simulations we conducted illustrated that even modest increases in transmission among Syrian refugees could result in a large increase in the incidence and cumulative total number of infections in the absence of interventions.

In conclusion, while the young age structure of the Syrian refugee population might play a protective role against the scale and disease-burden severity of a potential COVID-19 epidemic, the epidemic potential due to several vulnerability factors warrants an immediate response in this population group. Local and international actors are required to mobilize and coordinate efforts to prevent the transmission of COVID-19, and to mitigate its impact amongst the vulnerable refugee populations globally.

## Background

Since the start of the conflict in 2011, over 5.5 million people have fled Syria seeking refuge in neighboring countries [1]. Lebanon hosts the highest per capita number of refugees worldwide with approximately one million officially registered Syrian refugees [1]. Only 20% of those have legal residency, which complicates an already challenging situation in terms of housing, securing livelihood, and access to health care, among others [2]. About half of households live in extreme poverty and a large fraction continue to live in substandard settings [2]. Due to Lebanon’s non-encampment policy, Syrian refugees are widely dispersed throughout the country, both in urban and rural areas. The majority live in residential (69%) and non-residential (11%) structures in the host community, while the remaining 20% reside in informal tented settlements [2].

The protracted hosting of large refugee populations has placed additional strains on a middle-income country like Lebanon with ongoing political turmoil, unstable economic situation, and a fragmented, highly privatized, and under-resourced health care system. Since October 2019, Lebanon has been witnessing a popular uprising amidst an unprecedented economic crisis, which was further exacerbated by the emergence of the coronavirus disease 2019 (COVID-19) pandemic. In fear of a rapidly growing epidemic for which the health care system is largely unprepared, the government imposed early population lockdown which successfully suppressed the epidemic until July 2020 when infection spread resurged following rapid easing of restrictions. The August 4 Beirut port blast, which shattered the city and caused a large number of casualties, added further complexity to an already fragile situation. Several hospitals with large COVID-19 units were destroyed and the others were flooded with the injured, overstretching health care infrastructure and capacity. As of February 28, 2021, there were over 375,000 confirmed COVID-19 cases and 4,692 deaths, with hospitals in the country working near or at full capacity.

Despite substantial scale-up of the country’s testing capacity over time, testing of Syrian refugees has remained limited. As of July 10, 125 positive cases were confirmed among Syrian refugees, all of whom were identified in urban areas through contact tracing in clusters that were found among the local population [3]. Since the majority of the refugees live in the host community, their level of exposure is high due to close interaction with the wider population who is currently experiencing large epidemic expansion with a positivity rate close to 15%. Except for one national campaign conducted by UNHCR in informal settlements and collective shelters in June and where no cases were identified [3], there is no systematic testing of refugees living in these settings. While they may be relatively shielded due to lower contact with the host community and higher concentration of humanitarian efforts in the settlements, their living conditions create favorable context for a potentially large outbreak if the virus is introduced into these settings.

Refugees worldwide have been shown to have an increased vulnerability to infectious disease outbreaks compared to host populations [4], and COVID-19 is expected to be no exception. Despite some recent commendable efforts by UNHCR in scaling up preparedness plans [5], the response among the Syrian refugee population remains insufficient, particularly in terms of surveillance. In this commentary, we use quantitative insights of transmission dynamics to outline risk and contextual factors that may modulate vulnerability to potentially large COVID-19 epidemics among Syrian refugees in Lebanon.

## Vulnerabilities among refugees

### Risk factors directly impacting transmission dynamics

**Fig. 1:**
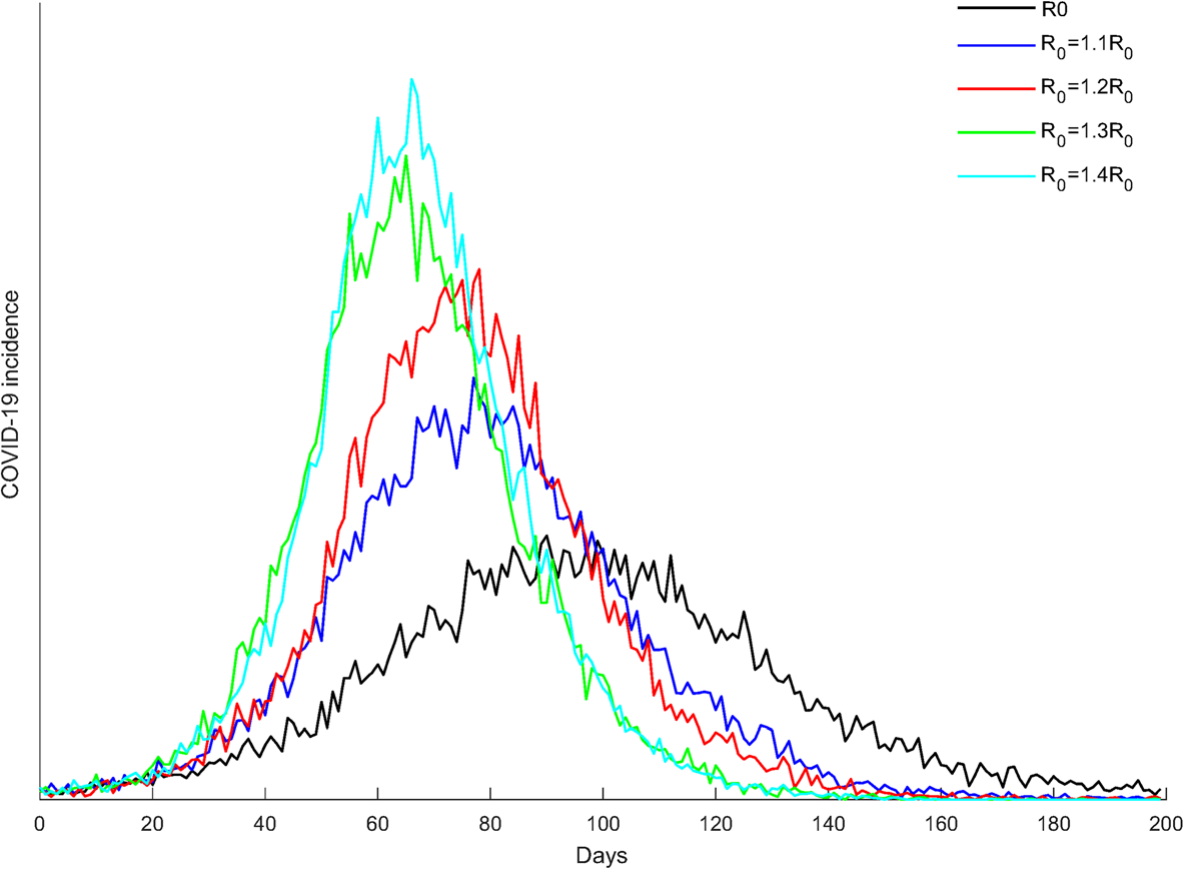
Mathematical modelling simulation of a COVID-19 epidemic in a refugee population assuming increase in the basic reproductive number (R_0_) by 10, 20, 30, and 40% compared to baseline

In an epidemic, an important parameter providing an indication of the potential spread of an infection is the basic reproductive number (R_0_) - defined as the average number of infections generated by one infected individual in a fully susceptible population in the absence of public health interventions. R_0_ can be expressed simplistically as: *R*_0_ = *c* × *p* × *D* where *c* is the contact rate, *p* is the probability of transmission per contact, and *D* is the duration of infectiousness. To illustrate the impact of the basic reproductive number on the extent of infection spread and, hence, the scale of the epidemic in any given population, we conducted mathematical modelling simulations of COVID-19 epidemics based on data from China. The methodology used to generate these scenarios is described in detail in another publication [6]. The simulations show that relatively modest increases in R_0_ by 10, 20 and 30% result in a large increase in the incidence and cumulative total number of infections in the absence of interventions (Fig. 1). Such an increase in R_0_ in an eventual epidemic in the Syrian refugee population in Lebanon is plausible for several reasons described below. The link of each one of these risk factors to R_0_ parameters is further summarized in Table 1.

1. The physical environment in which refugees reside may be one of the key factors that increase the likelihood of a large outbreak upon virus introduction. Syrian refugees have an average household size of five individuals, with 23% having more than seven members [2]. One-third of households are overcrowded, defined as having less than 4.5m^2^/person; and this proportion is highest in informal tented settlements at 46% [2]. Here, tents are usually packed tightly together, increasing the likelihood of contact between households. Consequently, physical distancing within crowded households is virtually impossible, and there is potentially a high rate of social contacts between refugee households, especially in informal tented settlements. Crowding impacts all three parameters of the R_0_ equation: a lack of physical distance between individuals increases the probability of transmission (*p*); densely populated shelters increase the contact rate (*c*); and inability to isolate (or quarantine) for those infected (or exposed) effectively increases the duration of infectiousness (*D*).
2. Inadequate access to clean water precludes the recommended prevention measures of hand/face washing and hence is likely to increase the transmission probability per contact (*p*). It has been estimated that 26% of Syrian refugee shelters in Lebanon lack basic sanitation services, which increases to about 40% in informal settlements [2]. On average, water supply costs US$48 US per household per month, a sizeable fraction of the average per capita weekly income of US$70 [2].
3. Inadequate access to and use of protective measures such as masks remain a concern among the refugee population due to low levels of awareness, low perceived risk of infection, cost considerations, and other factors. This puts refugees at higher risk of contracting the infection by increasing the transmission probability per contact (*p*). With mounting evidence on efficacy, universal mask wearing by the public has become the recommended policy to reduce transmission of COVID-19 [7–9], especially given the role of asymptomatic transmission. While humanitarian agencies have distributed hygiene kits including masks and soap bars, these efforts remain limited to refugees living in informal tented settlement and collective shelters [5], leaving out refugees in residential settings who have the highest rates of interaction with the host community.
4. While the infection does not discriminate between host populations or refugees, differential access to health care may impact the dynamics of the epidemic or its disease burden implications in the two populations. Outside of the ongoing epidemic, Syrian refugees experience numerous barriers to health care, such as cost, perceived low prioritization, and stigmatization which result in unmet health care needs [10]. It is estimated that in 2019, 13% had no access to any primary care and 24% did not have access to secondary healthcare [2], which may preclude diagnosis and isolation (hence effectively increasing *D*), and might contribute to further transmission to members of the households or wider community. Also, Lebanon already has an under-resourced health system and overall weak surveillance system with limited laboratory and testing facilities that may be prioritized for the host population. By the end of the first major lockdown on April 27, about 26,000 tests had been completed, none of which were among Syrian refugees. The first, and so far only, national testing campaign in informal settlement took place in June [3], 3 months into the pandemic and before the surge in cases started in the country. Lack of testing has direct implications for the cycle of diagnosis and isolation, and hence would increase R_0_ by effectively increasing the duration of infectiousness (*D*).
5. The level of awareness of symptoms and recommended course of action in case of onset of symptoms or exposure is yet another factor that may impact R_0_ by increasing the duration of infectiousness (*D*), given the delay to isolate or quarantine. A rapid response survey among a sample of refugees in informal tented settlements revealed that misconceptions about the infection are prevalent and that the majority of the respondents lacked knowledge on the national protocol and what to do in case of possible exposure, suspicion of infection, or onset of symptoms [11]. Lack of communication, poor access to reliable and accessible sources of information, and circulation of fake news and rumors are likely to delay refugees from recognizing symptoms and hence from presenting to health care or isolation.

**Table 1:** Vulnerability risk factors among Syrian refugees in Lebanon and their impact on the basic reproductive number (R_0_)

Risk factor	Consequence	Effect on R_**0**_ parameters
Crowded physical environment	• Increased contacts between households	• Increase in the contact rate (*c*)
	• Increased contacts within households	• Increase in the contact rate (*c*)
	• Short physical distance between members of a household	• Increase in the probability of transmission (*p*)
	• Inability to isolate or quarantine	• Increase in the duration of infectiousness (*D*)
Inadequate access to clean water	• Precludes hand/face washing and surfaces cleaning	• Increase in the probability of transmission (*p*)
Inadequate access to protective measures such as masks	• Increased spread of respiratory droplets	• Increase in the probability of transmission (*p*)
Inadequate access to health care	• Precludes diagnosis and isolation	• Increase in the duration of infectiousness (*D*)
Inadequate community awareness	• Delay to isolate or quarantine	• Increase in the duration of infectiousness (*D*)

### Contextual factors

In addition to the above factors that directly impact the basic reproductive number R_0_, there are several contextual factors that may increase the vulnerability of Syrian refugees in Lebanon to epidemic spread. For example, those caught violating curfew measures could be subject to legal measures including confiscation of identification [12]. Stigma and fear of arrest, deportation and loss of legal status exacerbate a lack of healthcare-seeking behavior and hence could contribute to further spread. Syrian refugees in Lebanon live under intense economic pressures - 69% live below the poverty line (<US$3.84/person/day), and 51% are extremely poor (Survival and Minimum Expenditure Basket <US$2.8/person/day) [2]. This will affect their ability to purchase masks, disinfecting products, and other hygiene items. The inability to cover even subsidized medical fees and transportation fees are additional barriers to seeking healthcare in case of exposure or onset of symptoms. Half of the refugees in the rapid response survey cited transport fees as a barrier to accessing medical care [11]. While COVID-19 testing is performed for free at the governmental hospital, refugees who might be reluctant to present for fear of stigma and discrimination would not be able to afford to test at a private laboratory at a fee of US$100.

In addition to increased risk of COVID-19 infection due to their living conditions, Syrian refugees in Lebanon have a relatively high prevalence of non-communicable diseases which were shown to be associated with increased risk of developing severe disease and death among those infected with COVID-19 [13, 14]. Half of the Syrian refugee households in Lebanon report having at least one member living with at least one non-communicable disease [15]. The prevalence, among the general refugee population and those aged more than 60, of hypertension is reported at 21 and 60% respectively, of cardiovascular disease at 11 and 30% respectively, and of diabetes at 10 and 47% respectively [10].

## Conclusions

In conclusion, Syrian refugees in Lebanon have several vulnerability factors that directly or indirectly affect COVID-19 transmission dynamics, and increase their risk of potentially large outbreaks (Fig. 1). These factors related to crowding, inadequate water supply and sanitation, inadequate access to health care, low prioritization from the host nation, stigma and fear of legal consequences, among others, are also common, to Syrian refugees in neighboring countries and to most refugee populations worldwide.

On the other hand, evidence is mounting regarding the protective effect of younger age on COVID-19 transmission dynamics [16–18]. It is predicted that settings with predominantly younger age cohorts and sizable children populations may experience smaller and relatively slow epidemics, while settings with sizable adult and/or elderly populations are likely to experience large and rapid epidemics in the absence of interventions [17]. With only 2% of the Syrian refugees worldwide being over 60 years of age (2.6% in Lebanon), and as much as 45% being less than 18 years (55% in Lebanon) [1], the age structure of this population may be favorable for a smaller epidemic. The younger demographic profile is also a protective factor for the burden of severe disease and mortality among those infected [6, 16, 17]. However, it is not yet clear whether the favorable age structure of refugees will outweigh the other risk factors that modulate high transmission dynamics within refugee populations. Mathematical modelling analyses taking into consideration these opposing factors would be able to predict the true scale and burden of COVID-19 epidemics in specific refugee populations.

However, even if the younger age of refugee populations may play a protective role, this should not lead to complacency. The infection burden in terms of morbidity and mortality has been shown to be not insignificant even among younger age groups. For example, in the US, there was a 19% increase in all-cause mortality among adults aged 25 to 44 years during the pandemic, and COVID-19 accounted for about 40% of this excess mortality [19]. If a COVID-19 epidemic is established among refugees, it would still cause a heavy toll given all the vulnerability factors and the resource-poor healthcare infrastructure available to this population. Therefore, to prevent exacerbation of the current humanitarian crisis and catastrophic consequences, local and international actors and stakeholders should urgently mobilize and coordinate efforts to prevent the transmission of COVID-19, and mitigate its impact amongst the vulnerable refugee populations globally [20]. Special consideration also needs to be given to refugee populations when prioritizing vaccine deployment. In Lebanon, UNHCR has recently strengthened its preparedness to respond to COVID-19 by implementing a strategy integrated within the national response. This includes setting up isolation facilities, expanding hospital capacity, covering all testing and treatment costs for refugee patients, as well as engaging communities and raising awareness [5]. These efforts need to be sustained and expanded as the country is currently experiencing major epidemic expansion which may have devastating consequences if it affects the refugee population.

## Data Availability

Not applicable.

## Abbreviation

COVID-19: Coronavirus disease 2019
